# TIPS: A Framework for Text Summarising with Illustrative Pictures

**DOI:** 10.3390/e23121614

**Published:** 2021-11-30

**Authors:** Justyna Golec, Tomasz Hachaj, Grzegorz Sokal

**Affiliations:** Institute of Computer Science, Pedagogical University of Krakow, 2 Podchorazych Ave, 30-084 Krakow, Poland; justyna.golec@up.krakow.pl (J.G.); grzegorz.sokal@up.krakow.pl (G.S.)

**Keywords:** deep learning, image-text matching, illustrative images, semantic multi-modal matching, image-text similarity, natural language processing, voting schema

## Abstract

We propose an algorithm to generate graphical summarising of longer text passages using a set of illustrative pictures (TIPS). TIPS is an algorithm using a voting process that uses results of individual “weak” algorithms. The proposed method includes a summarising algorithm that generates a digest of the input document. Each sentence of the text summary is used as the input for further processing by the sentence transformer separately. A sentence transformer performs text embedding and a group of CLIP similarity-based algorithms trained on different image embedding finds semantic distances between images in the illustration image database and the input text. A voting process extracts the most matching images to the text. The TIPS algorithm allows the integration of the best (highest scored) results of the different recommendation algorithms by diminishing the influence of images that are a disjointed part of the recommendations of the component algorithms. TIPS returns a set of illustrative images that describe each sentence of the text summary. Three human judges found that the use of TIPS resulted in an increase in matching highly relevant images to text, ranging from 5% to 8% and images relevant to text ranging from 3% to 7% compared to the approach based on single-embedding schema.

## 1. Introduction

The development of deep neural networks (DNN) has revolutionised issues related to the analysis of images and natural language processing [1]. The ability to generate feature vectors (embedding) from both images and texts has greatly facilitated the semantic analysis of these media. Especially interesting are issues of image-text matching to determine the semantic similarity between them. Image-text matching is an important multi-modal task with a wide range of applications [2]. Research in this area using deep neural networks is relatively new, and many of the relevant results have been published in work from within the last three years.

### 1.1. State-of-the-Art on Image-Text Matching

Modern methods of comparing and matching text to images are based almost exclusively on deep neural networks [3,4]. We can distinguish several basic issues that determine how to select machine learning methods to make this process efficient and effective. The first is the appropriate choice of architecture and scale of the neural network. Convolutional Neural Networks (CNN) [5] are commonly developed on a fixed resource budget, and then scaled up for better accuracy if more resources are available. Tan et al. [6] systematically studied model scaling and identify that carefully balancing network depth, width, and resolution can lead to better performance. They propose a new scaling method that uniformly scales all dimensions of depth/width/resolution using a simple yet highly effective compound coefficient. Due to the fact that pre-training is a dominant paradigm in computer vision, [7] image features are commonly retrieved using well-established CNN architectures. The next issue is the application of the image search algorithm. Image Search is a fundamental task playing a significant role in the success of a wide variety of frameworks and applications [8]. An important method to compare semantic similarity between text and images is CLIP Contrastive Language-Image Pre-Training). Conde et al. [9] proposed that a CLIP approach be used for training a neural network on a variety of art images and text pairs, being able to learn directly from raw descriptions about images, or if available, curated labels. Visual attention not only improves the performance of image captioners, but also serves as a visual interpretation to qualitatively measure the caption rationality and model transparency [10]. The use of the CLIP method enabled us to search for illustrative images in which the images do not have ready-made annotations (compare with Joshi et al. [11]). Huang et al. [12] developed an image-text matching approach using a bi-directional spatial-semantic attention network which leverages both the word to regions relation and visual object to words relation in a holistic deep framework for more effective matching. Image-text matching for short (one-sentence) texts has been realised in practice in the Sentence Bert (SBERT) [13] algorithm. It uses the language module BERT [14]. BERT is a language representation model while SBERT generates feature vectors from both image and text and compares them using CLIP. SBERT was developed to optimise previously used solutions such as InferSent [15], Universal Sentence Encoder [16] and SentEval [17]. Vision models trained on multi-modal data sets can benefit from the wide availability of large image-caption data sets. According to [18] CLIP proved to be a reliable method in multi-modal semantic task solving.

Additionally, the latest research findings in the topics of this article can be found in survey papers. Zhu et al. [19] and Rudinac et al. [20] discuss the latest achievements in story summarising. Baltrušaitis et al. [21] and Guo et al. [22] survey the recent advances in multimodal machine learning and present them in a common taxonomy. In reviews [23,24], Gao et al. and Ramachandram et al. present a survey on deep learning for multimodal data fusion to provide readers, regardless of their original community, with the fundamentals of a multimodal deep learning fusion method and to motivate new multimodal data fusion techniques of deep learning. With the rapid growth of social media, users post large volumes of data in various modalities such as text, image, audio, and video. In surveys [25,26], Huddar et al. and Soleymani et al. define sentiment, sentiment analysis, states problems and challenges in multimodal sentiment analysis and finally review some of the recent computational approaches used multimodal sentiment analysis. The Multimodal data-driven approach has emerged as an important driving force for smart healthcare systems. Cai et al. [27] provide a comprehensive survey of existing techniques which include not only state-of-the-art methods but also the most recent trends in the field.

### 1.2. Study Motivation

In this paper, we propose an algorithm to generate graphical summarising of longer text passages using a set of illustrative pictures, which we refer to as Text Summarising with Illustrative Pictures (TIPS). TIPS is an algorithm using a voting process that uses the results of individual “weak” algorithms to produce a single result. The proposed method includes a summarising algorithm that generates a digest of the input document. Then, a sentence transformer performs text embedding and a group of CLIP similarity-based algorithms trained at image embedding finds semantic distances between the images in the illustration image database and the input text snippets. This is followed by a voting process that extracts the most similar images to the text. Both the TIPS algorithm scheme, the voting process algorithm, and the methodology for evaluating and comparing our algorithm with other image-text matching methods are original achievements presented in this work. Our proposed method matches illustrative images to each sentence that was generated by the summarizer algorithm independently. Therefore, the ability of our algorithm to summarize text using a set of illustrative images relies on the power of the summary-generating algorithm only.

## 2. Material and Methods

### 2.1. Text and Image Processing Framework

#### 2.1.1. Text Summarising

Single-document summarising is the task of automatic generation of a shorter document version while retaining its most important information [28]. Currently, neural network-based algorithms trained on relevant language corpora are used to generate text summaries [29,30,31]. Text summarising typically use a variety of language representation models. Among the most popular and effective models of this type is the BERT (Bidirectional Encoder Representations from Transformers) algorithm [14]. BERT is also based on a neural network, which, among other things, is designed to model the strong connections between words of a language in order to find their representation, which will then be used to generate a summary. Unlike other solutions of this type, BERT is designed to pretrain deep bidirectional representations from unlabelled text.

Text summarizers currently use summarising-specific neural architectures to enhance document-level features. We decided to use the architecture proposed in [28] called BERTSUM. Suppose we have a text $X=[x_{1},x_{2},\ldots ,x_{n}]$, where $x_{i},i\in \left[1,n\right]$ are sentences of this text. The task of the summarizer is to select n out of the m sentences that this text consists of (n⩽m). In this algorithm, a feature vector is generated for each sentence that is part of the text. Then the whole text is processed by Inter-sentence Transformer:

(1) $$\left\{\begin{array}{c} p_{0}=B\\ p_{i}=LN\left(p_{i-1}+AO\left(p_{i-1}\right)\right)\\ p_{i}^{\prime }=LN(p\left(i\right)+FFN\left(p\left(i\right)\right) \end{array}\right.$$

where: *B* is an output vector of BERT, LN is layer normalisation [32], AO is the attention operation [33] and FFN is feedforward neural network with depth *i*.

The final layer is a sigmoid classifier. The output from BERTSUM for each of the sentences included in the text generates a value between [0, 1]. The larger the value of the output signal from BERTSUM, the more confident the sentence should be in the summary. The obtained summary $S_{x}=\left[x_{j_{1}},...,x_{j_{m}}\right]$ is a subset of the original text *X* and there are m indices j∈{1,...,n}.

#### 2.1.2. Sentence Transformer

Sentence transformers are a group of methods for generating a vector of features that describe a sentence (sentence embedding) [34]. A feature of such descriptive vectors is that the algorithms that generate them minimise the distance between vectors of sentences that have similar semantic meaning. Sentence transformer algorithms use a language model to generate a feature vector from a given sentence, which is then the input argument to further processing. In practice, the sentence transformer uses a deep neural network that recalculates the input vectors so that a distance metric can be used to calculate the semantic distance between sentences.

The method proposed in [13] uses the BERT language model, which we described in Section 2.1.1. The output vectors from BERT are then processed with a deep architecture that uses the triplet loss function [35] for training. With triplet loss, the distance between embedding sentences that have similar semantic meaning is minimised during training and the distance between embedding sentences that have different semantic meaning is maximised.

#### 2.1.3. Image Embedding

Most common image embedding solutions are trained as models to classify large data sets of diverse images, for example ImageNet [36]. Networks of this type can also be used to generate feature vectors of images. In this case, the initial convolutional layers of the network are used, which generate a single, typically several hundred dimensional vector describing the input image. Classifier layers of the network are then not used. The most popular pre-trained implementations of convolutional deep neural networks are VGG16 [37], ResNet50 and its modifications [38], InceptionV3 [39] or MobileNet [40].

#### 2.1.4. Image-Text Matching

Image-text matching is a group of methods that allows evaluating the semantic similarity between text and image content by measuring this similarity using a given metric. Modern algorithms of this type use sets of methods that we discussed in earlier sections and have much in common with image classification problems. Image classification, which boils down to assigning an image to one of a predefined class, is a very well-studied and widely applied problem, as we pointed out in Section 2.1.3. The problem of determining image class can also be considered more broadly by using methods that will automatically generate descriptions of the contents of images. This is currently done using deep models that combine image embedding with the ability to generate descriptions using recurrent Long short-term memory (LSTM) neural layers such as [41,42,43]. A large survey on this field can be found at [44,45]. Thus, one could use such automatically generated image descriptions with sentences that have been generated by the text summarizer (see Section 2.1.1) to determine the similarity between the text and the various images we have, in order to select the image that minimises the given distance metric [46]. However, this approach has a major drawback: the quality of image descriptions generated by automatic algorithms is not yet perfect, and semantic comparison of texts is a complex issue. This results in a build-up of errors generated by both approaches, which can significantly affect the quality of the entire text-to-image matching method. For this reason, dedicated and specially trained solutions are used for image-text matching, whose individual components originate from the areas of natural language processing, text embedding and image embedding.

A Contrastive Language-Image Pre-training (CLIP) method based on neural network architecture has been is proposed in the paper [47] to evaluate the similarity between text and image. In order to perform CLIP, a similarity assessment algorithm learns a multi-modal embedding space by jointly training an image encoder and text encoder to maximise the cosine similarity of the image and text embedding. The utilised batch construction technique used in CLIP is the multi-class N-pair loss [48]. Image-text similarity is calculated as follows:

(2) $$\begin{array}{c} I_{e}=L2\left(I_{f}\circ W_{I}\right)\\ T_{e}=L2\left(T_{f}\circ W_{T}\right)\\ sim_{clip}=\left(I_{e}\circ T_{e}\right)\cdot e^{p} \end{array}$$

where: L2 is euclidean norm, ∘ is a dot product, $I_{f}$ is image features (embedding), $T_{f}$ is text features (embedding), $W_{I}$ is learned projection of image to embedding (to be trained during CLIP model fitting), $W_{T}$ is learned projection of text to embedding (to be trained during CLIP model fitting), *p* is a learning rate. The training procedure of CLIP is based on minimisation of cross entropy loss. CLIP utilises Transformer text encoder (embedder) [49] and image embedding algorithm (i.e., Vision Transformer image encoder (embedder) [50], ResNet etc.).

### 2.2. Data Sets

In this subsection we describe the data set that we have utilised in our research.

#### 2.2.1. Text Data Set

The text dataset was obtained from the Brunel University London website (https://brunel.figshare.com/articles/dataset/4000_stories_with_sentiment_analysis_dataset/7712540, (accessed on 27 September 2021)). The study used 426 short stories that are equal to or less than 1000 characters in length. Due to this we will call this data set Stories426. The collection consists of humorous stories or short tales with a moral. The authors include Aesop, Ambrose Bierce, James Baldwin or Kate Chopin. All texts have been summarised using a BERTSUM (see Section 2.1.1 for exact information), then divided into sentences. This data set was chosen because of its use of natural language and the variety of stories. An additional advantage, is that the sentences are written correctly in terms of style and language. Obtaining linguistically correct texts is more difficult with data from social media platforms because their users often use verbal abbreviations, e.g., ‘LOL’ which means laugh out loud, ‘U’ which is equivalent to ‘you’, do not use complete sentences or forget about linguistic correctness. For the stories used, the full story is described, which helps in creating a full illustration for the text. A person can also easily verify if the proposed images match the content they are paired with. Another advantage of this data set is that it is freely available to the public.

#### 2.2.2. Image Data Set

The image data set was built from nearly 25,000 (24,996) publicly available nature-themed images, sourced from the Unsplash platform (https://unsplash.com/ (accessed on 27 September 2021)). Embedding for this set of images was generated using several deep neural networks. We have used a 50-layer residual network (ResNet 50, RN50), 101-layer residual network (ResNet 101, RN101), and a four times scaled RN50 according to the EfficientNet scaling rule [6]. We also used the Vision Transformer image encoder mentioned in Section 2.1.3 in order to generate embedding for two of its architectures: ViT-B32 (ViT32) and ViT-B16 (ViT16). The Vision Transformer image encoder, in comparson to state-of-the-art DNN models we mentioned in Section 2.1.3, requires substantially fewer computational resources to train. We have used the pre-trained web weights provided at https://github.com/openai/CLIP/blob/main/clip/clip.py (accessed on 27 September 2021). The use of Transformer text embedding [49] and ViT32 image embedding is the same as the SBERT solution architecture [13].

### 2.3. Proposed Method for Text Summarising with Illustrative Pictures (TIPS)

**Figure 1:**
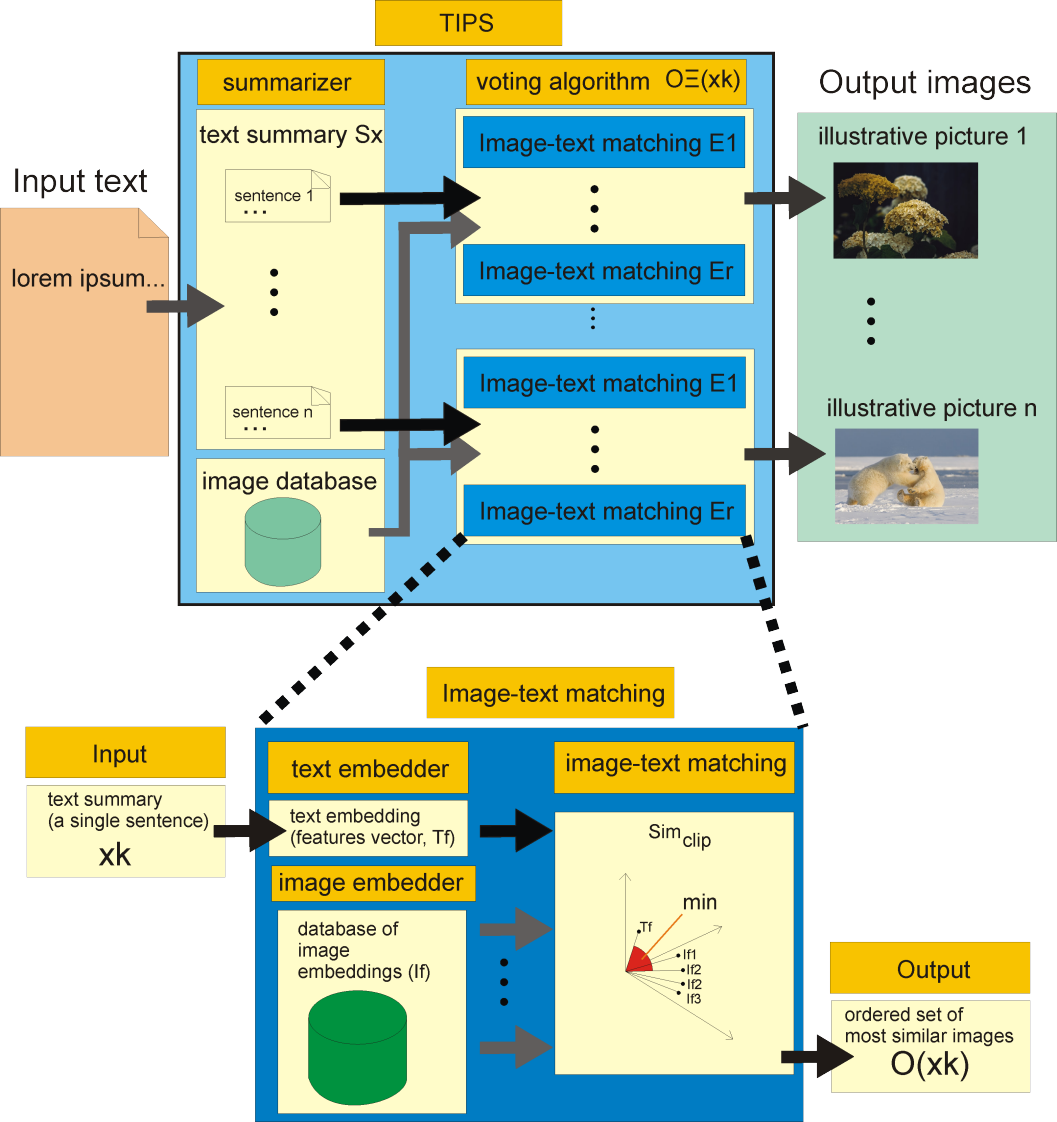
This figure presents the pipeline of the proposed algorithm for text summarising with illustrative pictures (TIPS) algorithm.

In Figure 1, we present the data processing pipeline of our proposed algorithm for generating a graphical text summary using illustrative images. It is an algorithm using a properly designed voting process that uses the results of individual “weak” algorithms to produce a single result.

The first step is to prepare a text summarizer using a summarising algorithm. For this purpose, we use the BERTSUM algorithm described in Section 2.1.1. Then, using the sentence transformer, we perform text embedding using the Transformer algorithm [47]. As a set of illustration images $O=\{o_{1},o_{2},...,o_{r}\}$ (*r* is image count of the data set that contains all potential illustrative images) should contain diversified set of images with a wide range of topics if the texts to be analysed are to cover a wide range of topics. If we assume that texts are to cover a narrower range of specialised topics we use a set of images that are characteristic to those very topics i.e., architecture, sport events etc.

Let us first consider the performance of a single image-text matching algorithm. The algorithm uses the given image embedding method (a suitable deep neural network, see Section 2.1.3). The input text is processed by the sentence transformer algorithm (see Section 2.1.2). The image and text feature vectors are used to train CLIP (see Section 2.1.4). This training only needs to be done once. Assuming that we have a given image database to serve as a source of illustrative images, we can perform an equal embedding of this entire database using image embedding, which is part of the given image-text matching method. For the given database, this process also takes place once.

After the text summary is performed, each sentence extracted from the text is processed by a set of image-text matching algorithms (see Section 2.1.4). Image-text matching is performed for each sentence in the text summary separately. For each summary, a vector is generated which coordinates are numbers that are proportional to the semantic similarity between the text and the illustration: see Equation (2). Each sentence will be represented by an illustration image for which the value of (2) reaches a maximum.

(3) $$\forall x_{k}\in S_{x}\to o_{l}:max_{l}\left(sim_{clip}\left(o_{l},x_{k}\right)\right)$$

Evaluation of the quality of image to text assignment is performed using similarity $sim_{clip}$.

Let:

(4) $$O\left(x_{k}\right)=ord\left(O,sim_{clip}\left(o_{l},x_{k}\right)\right)$$

be a set of images O ordered by $sim_{clip}$ between sentence $x_{k}$ and image $o_{l}\in O$ in descending order. In other words $O(x_{k})$ is a set of images where first element has the highest value of $sim_{clip}$ with $x_{k}$ and the last element has the smallest value of $sim_{clip}$ with $x_{k}$.

The ordering (4) depends on the image embedding E we have used. For this reason, we can write more generally:

(5) $$O^{E}\left(x_{k}\right)=ord\left(O,sim_{clip}^{E}\left(o_{l},x_{k}\right)\right)$$

Let $O^{E}{\left(x_{k}\right)}_{t}$ be an ordered subset of the set (5), consisting of *t* initial elements of $O^{E}\left(x_{k}\right)$. With *r* different embedding methods defined $Xi=E_{1},...,E_{r}$ we can propose an algorithm that will use (5) to create a voting scheme. The goal of this scheme will be to order the sets of images proposed by each embedding in Ξ order according to the sum of the normalised $sim_{clip}^{E}$:

(6) $$O^{\Xi }{\left(x_{k}\right)}_{t}=\overset{}{\underset{\Xi }{⋃}}O^{\prime E}{\left(x_{k}\right)}_{t}$$

In the case of $O^{\prime E}{\left(x_{k}\right)}_{t}$, the similarity between $x_{k}$ and $o_{l}$ is computed as:

(7) $$sim_{clip}^{\prime E}\left(x_{k},o_{l}\right)=\frac{sim_{clip}^{E}\left(x_{k},o_{l}\right)}{\sum_{s=1}^{t}sim_{clip}^{E}\left(O{\left(x_{k}\right)}_{s},o_{l}\right)}$$

That means that the similarities between $x_{k}$ and $o_{l}$ for a given E∈Ξ are divided by the sum of the similarities of the first p similarities between $O{\left(x_{k}\right)}_{s}$ and $o_{l}$ ordered according to (5). This operation is done to pseudo-normalise $sim_{clip}^{E}$ so that the individual orderings of $O_{t}^{\prime E}$ have comparable ranges of $sim_{clip}^{E}$ values. Thus, the ordered set $O^{E}{\left(x_{k}\right)}_{t}$ consists of the sum of the elements included in the individual ones of $O^{\prime E}{\left(x_{k}\right)}_{t}$. The criterion for ordering the images $o_{l}$ that are part of $O^{\prime E}{\left(x_{k}\right)}_{t}$ is the sum of the (7) that has been assigned to a given $o_{l}$ by the individual E∈Ξ. If any $O^{E}{\left(x_{k}\right)}_{t}$ does not contain $o_{l}$, then we assume that for this E $sim_{clip}^{\prime E}\left(x_{k},o_{l}\right)=0$.

In the simplest case where t=1 voting schema (6) works like majority voting schema. In case two or more images received the same number of votes as the most similar to the given text, one of these most similar items is returned randomly.

As the value of t increases, the voting algorithm (6) will propose $O^{\Xi }{\left(x_{k}\right)}_{t}$, which will contain an increasing number of proposed images. The order of these images might differs. Of course, it is always true that:

(8) $$O^{\Xi }{\left(x_{k}\right)}_{p}\subset O^{\Xi }{\left(x_{k}\right)}_{t+1}$$

This means that all images that are in the set $O^{\Xi }{\left(x_{k}\right)}_{t}$ for a given p are also in the set $O^{\Xi }{\left(x_{k}\right)}_{t+1}$.

The above Equation (7) defines our proposed text summarising with illustrative pictures (TIPS) method. In summary, for the text *X*:

(9) $$X\overset{TIPS}{\to }\left\{o_{k_{1}},\ldots ,o_{k_{n}}\right\}$$

We can also write (9) as:

(10) $$TIPS\left(X\right)=\left\{o_{k_{1}},\ldots ,o_{k_{n}}\right\}$$

Summarizer turns paragraph text into sentences and then passes the tokenized sentences to the BERT model for inference to output embedding. That embedding is then clustered with K-Means in order to select sentences that are closest to the centroid. Those closest sentences are candidates for summary [51]. Due to this fact the summary consists of sentences that are already present in the original text.

The principles of the CLIP method in practice limit its effectiveness to single sentences, so it would be ineffective to use it for the entire text [47]. It is possible that embedding of the certain sentences will not create “spherical” clusters with representative centroid. In that scenario the “centre” sentence might be not representative for the whole text, however already published papers proved for some extend that application of K-means clustering for finding most important sentences resulted in relatively high ROUGE scores in comparison to other approaches [52,53,54]. Due to this fact we did not evaluate the quality of the obtained summary.

### 2.4. Evaluation and Comparison of Image-Text Matching Algorithms

Evaluation of image-text matching in terms of semantic similarity of large texts is a very difficult issue because we do not have a data set for which there would be a ground truth. Additionally, if we want to compare results obtained by two or more different image-text matching algorithms that use the clip similarity measure, we need to remember that each of these algorithms is trained independently, and it would be incorrect to compare these similarity measures directly. For this reason, we have proposed a number of coefficients that are useful for evaluating and comparing image-text matching algorithms.

While we cannot directly compare $sim_{clip}$ values between methods, we can examine the semantic distances returned between successive recommended images:

(11) $$\left\{\begin{array}{c} m_{1,2}\left(x_{k}\right)=sim_{clip}\left(O{\left(x_{k}\right)}_{1},x_{k}\right)-sim_{clip}\left(O{\left(x_{k}\right)}_{2},x_{k}\right)\\ m_{1,3}\left(x_{k}\right)=sim_{clip}\left(O{\left(x_{k}\right)}_{1},x_{k}\right)-sim_{clip}\left(O{\left(x_{k}\right)}_{3},x_{k}\right) \end{array}\right.$$

where $O{\left(x_{k}\right)}_{1}$ is a first element in $O(x_{k})$, $O{\left(x_{k}\right)}_{2}$ is a second element in $O(x_{k})$ etc. Due to this $m_{1,2}\left(x_{k}\right)$ is a difference between $sim_{clip}$ value of the most similar image and the second most similar image. A high value of this index may indicate that the image data set is diverse as well as that the selected most similar image is significantly more similar to the text than the other images that are in the image set while using certain E. The average value of these indexes can also be counted for the entire test set TS:

(12) $$\left\{\begin{array}{c} \bar{M_{1,2}}=\frac{\sum_{X\in TS}^{}\sum_{x_{k}\in S_{X}}^{}m_{1,2}\left(x_{k}\right)}{\#(\sum_{X\in TS}^{}n_{X})}\\ \bar{M_{1,3}}=\frac{\sum_{X\in TS}^{}\sum_{x_{k}\in S_{X}}^{}m_{1,3}\left(x_{k}\right)}{\#(\sum_{X\in TS}^{}n_{X})} \end{array}\right.$$

where $\#(\sum_{X\in TS}^{}n_{X})$ is the cardinal number of the set, that are summaries of each text *X* contained in the set TS. Equation (11) are statistics for a single text. Equation (12) are statistics for the entire set ST.

We can also examine the common part of the set of recommended illustration images for all algorithms included in Ξ:

(13) $$In\left(\Xi ,t\right)=\frac{\#(\forall x_{k}\in S_{x}:\left({⋂}_{\Xi }^{}O^{\prime E}{\left(x_{k}\right)}_{t}\right)}{n}$$

as well as the sum of such sets:

(14) $$Un\left(\Xi ,t\right)=\frac{\#(\forall x_{k}\in S_{x}:\left({⋃}_{\Xi }^{}O^{\prime E}{\left(x_{k}\right)}_{t}\right)}{n}$$

Obtained values inform us how much variation there is in the t first recommendations of each of the algorithms included in Ξ.

Another statistic is the cardinality of the set composed of common part of set of images recommended by (6) for t and set of images recommended by (6) for t+1 divided by the number of all summaries TIPS(X).

(15) $$V\left(\Xi ,t\right)=\frac{\#(\forall x_{k}\in S_{x}:sim_{clip}\left(O^{\Xi }{\left(x_{k}\right)}_{t}1,x_{k}\right)=sim_{clip}\left(O^{\Xi }{\left(x_{k}\right)}_{t+1}1,x_{k}\right))}{n}$$

The above equation determines how much successive votes are consistent with each other, and can be thought of as a way of determining the stability of the voting process as well as the consistency of the recommendations returned by each component $O^{\prime E}{\left(x_{k}\right)}_{t}$. It can be seen that V(Ξ,t)∈[0,1]. The value of V(Ξ,t) equals 0 when:

(16) $$\forall x_{k}\in x_{k}:sim_{clip}\left(O^{\Xi }{\left(x_{k}\right)}_{t}1,x_{k}\right)\neq sim_{clip}\left(O^{\Xi }{\left(x_{k}\right)}_{t+1}1,x_{k}\right)$$

The value of V(Ξ,t) equals 1 when:

(17) $$\forall x_{k}\in x_{k}:sim_{clip}\left(O^{\Xi }{\left(x_{k}\right)}_{t}1,x_{k}\right)=sim_{clip}\left(O^{\Xi }{\left(x_{k}\right)}_{t+1}1,x_{k}\right)$$

Counterintuitively, the phenomenon where V(Ξ,t)=1 is not necessarily an advantageous situation. This situation means that every E∈Ξ always returns an identical first image recommendation, which may imply little diversification in the embedding methods used.

A set of following statistics is also worth investigating because they report the effect of the t parameter on the performance of the TIPS algorithm (this will be discussed in Section 4):

(18) $$\left\{\begin{array}{l} sim_{min}\left(X\right)=min\left(TIPS\left(X\right)\right)\\ sim_{max}\left(X\right)=max\left(TIPS\left(X\right)\right)\\ sim_{mean}\left(X\right)=mean\left(TIPS\left(X\right)\right)\\ sim_{med}\left(X\right)=med\left(TIPS\left(X\right)\right) \end{array}\right.$$

where: min is minimal, max is maximal, mean is mean and med is median value of $sim_{clip}$ among all image-text matching calculated by TIPS.

We have also added an evaluation of the obtained image recommendation results by three human judges. One of the evaluators was a co-author of this paper, (T.H.) and two others were persons not directly related to the research presented in this paper and without a background in computer science. To each judge, the computer program presented a sentence generated by the summary algorithm and six illustrative images selected by TIPS and using non-voting single-embedding schema (4) in which E was ViT16, ViT32, RN50, RN101, and RN50x4. Those six illustrative images were displayed in a random order so as to eliminate the situation where the judge assigned some meaning to the order of the images. Judges were informed of the purpose of the study and that the images would be presented in random order. Each judge independently assessed the relationship between the text and each of the six illustrative photographs using a three-point judging rating scale (JS) in the range [0–2] according to the subjective impressions. Each judge made evaluation separately without contacting two others. This approach is similar to [55]:

- JS = 0: the image is not relevant to the text;
- JS = 1: the image is relevant to the text;
- JS = 2: the image is highly relevant to the text.

In the next section, we will present the evaluation results of our method on the data sets discussed in Section 2.2.1.

## 3. Results

In order to find illustrative images for each $S_{X}$ according to the TIPS method described in Section 2.3, we have prepared an implementation of the proposed solution.

Our proposed method for finding illustrative images for text was implemented in Python 3.5. In order to generate $S_{X}$ for each of the short stories included in the Stories426 collection described in Section 2.2.2, we implemented the method described in Section 2.1.1 based on the solution proposed by Miller [51]. For this purpose we used the libraries spaCy 3.1, Transformers 4.1, NeuralCoref 4.0, Summarizer 0.0.7, Sentencepiece 0.1.96, pytorch_pretrained_bert 0.6.2. Computations were performed on the Google Colab platform using Torch 1.6 computational libraries.

Embedding of text was done using the Transformer algorithm [49] using the implementation of https://sbert.net/docs/package_reference/SentenceTransformer.html (accessed on 27 September 2021). Embedding of images was done using the methods described in Section 2.1.3 and CLIP similarity computation was done using the algorithm described in Section 2.1.4. We used Pytorch 1.7 and Torchvision cudatoolkit 11. The source codes and data sets of the programs we prepared can be downloaded from https://github.com/JusMia/TIPS (accessed on 27 September 2021).

We have made recommendations using the illustrative images presented in Section 2.2.2 for each text that was in the Stories426 using the TIPS algorithm utilising the voting schema (6). We used the image embedding methods ViT16, ViT32, RN50, RN101, and RN50x4 in the voting algorithm $O^{\Xi }{\left(x_{k}\right)}_{t}$ (6). We performed calculations for *p* values with an interval of [1,100]. For the purposes of evaluation we have also calculated illustrative image recommendations using non-voting single-embedding schema (4), in which E was ViT16, ViT32, RN50, RN101, and RN50x4.

**Figure 2:**
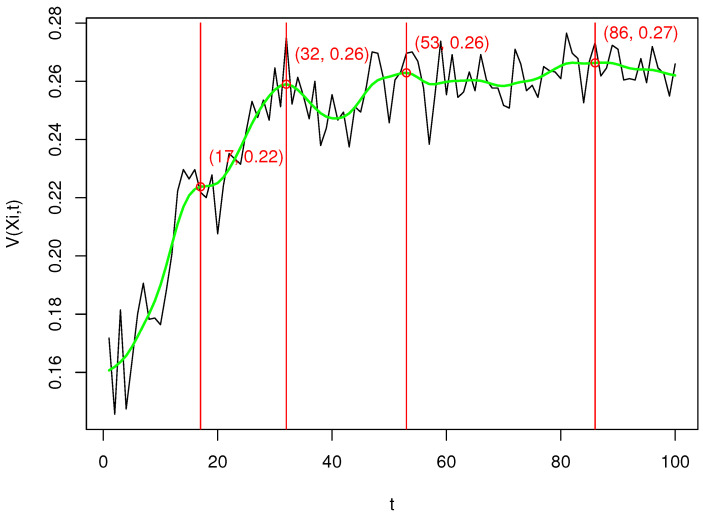
Plot of V(Ξ,t) values for different *p* of the TIPS algorithm. The individual results have been connected by the segments highlighted in black. We smoothed the resulting plot using spline approximation. We have marked the spline in green. In red we have marked four local maxima of the smoothed function V(Ξ,t).

Figure 2 shows the values of V(Ξ,t) for different p. The separate results were connected by the segments marked in black. We smoothed the resulting plot using spline approximation. We have marked the spline in green. In red we marked four local maxima of the smoothed function V(Ξ,t). For the corresponding values of *p* we will perform a more detailed analysis later on.

In Table 1, we presented values of coefficients $\bar{M_{1,2}}$ and $\bar{M_{1,3}}$ (12) for recommendations $O^{E}\left(x_{k}\right)$ where embedding E used algorithms ViT16, ViT32 (equivalent to the SBERT method), RN50, RN101, RN50x4, and our proposed TIPS algorithm with t-values equal to 17, 32, 53, and 86, which were the local maxima detected previously (see Figure 2).

**Figure 3:**
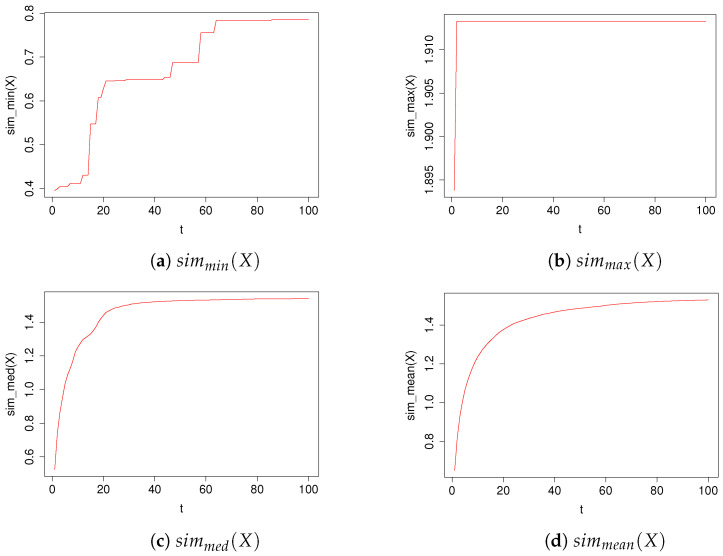
Performance of the TIPS algorithm for different values of the parameter t.

**Figure 4:**
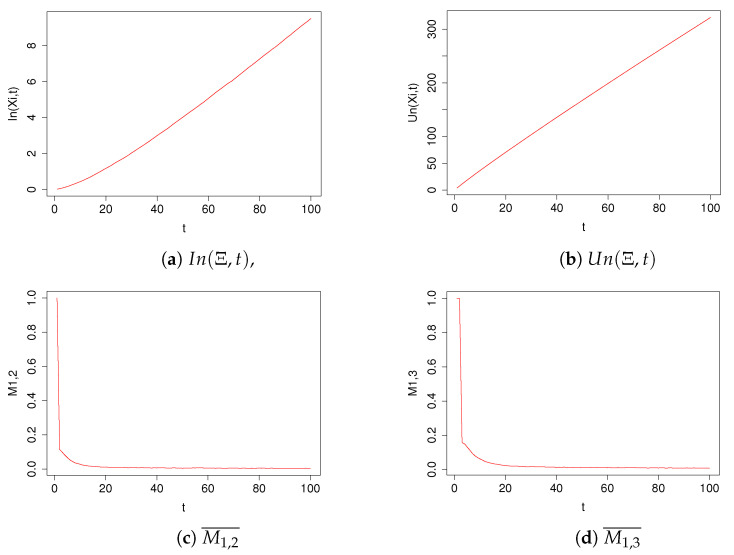
Performance of the TIPS algorithm for different values of the parameter t.

In plots in Figure 3 we have shown the values of the coefficients $sim_{min}\left(X\right)$, $sim_{max}\left(X\right)$, $sim_{mean}\left(X\right)$, $sim_{med}\left(X\right)$ for the TIPS algorithm with parameter values *t* in the range [1,100]. In plots in Figure 4 we have shown the values of the coefficients In(Ξ,t), Un(Ξ,t), $\bar{M_{1,2}}$ and $\bar{M_{1,3}}$ for the TIPS algorithm with parameter values *t* in the range [1,100].

In Table 2, we have presented the exact values of the coefficients of the TIPS algorithm shown in Figure 3 and Figure 4 for the selected t values.

**Figure 5:**
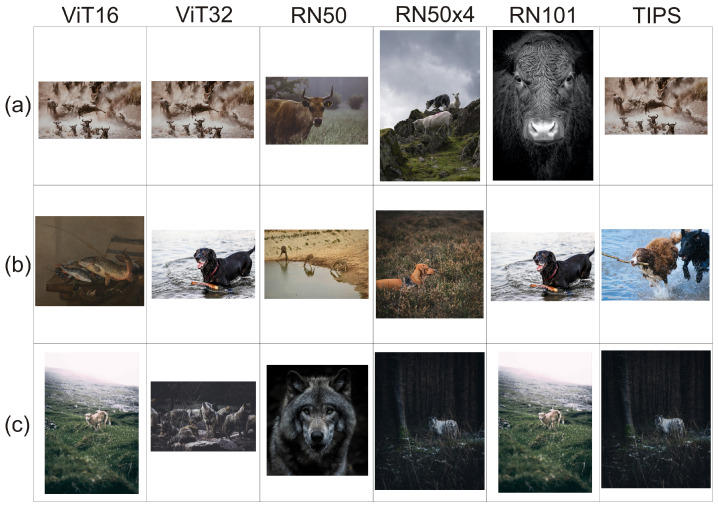
Sample illustrative image recommendation results for three sample text summaries $x_{k}$. The texts are: (**a**) “A Bull once escaped from a Lion by entering a cave which the Goatherds used to house their flocks in stormy weather and at night”; (**b**) “A fine hide makes an excellent meal for a hungry Dog, but the water was deep and the Dogs could not reach the hides from the bank”; (**c**) “The next day, dressed in the skin, the Wolf strolled into the pasture with the Sheep”.

In Figure 5 we have shown example of the illustrative image recommendation results for three example text summaries $x_{k}$. Images are calculated as $O^{E}{\left(x_{k}\right)}_{1}$ for the embedding algorithms ViT16, ViT32, RN50, RN50x4, RN101 and $O^{\Xi }{\left(x_{k}\right)}_{1}$ for TIPS, p=32.

**Figure 6:**
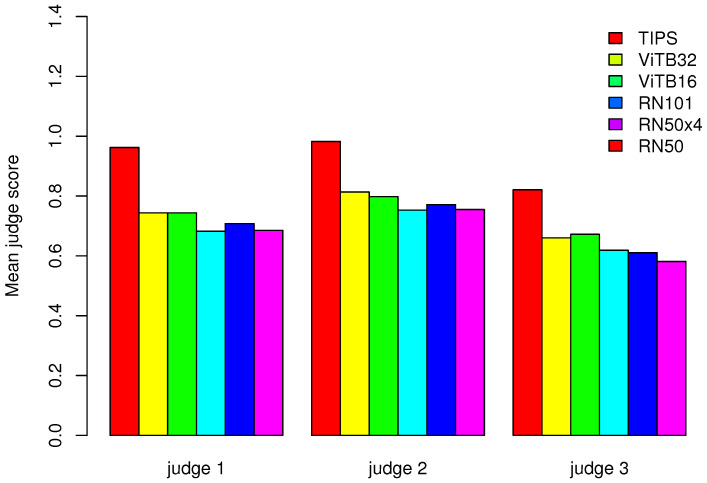
JS averaged over each illustrative image recommendation algorithm (see Table 3).

**Figure 7:**
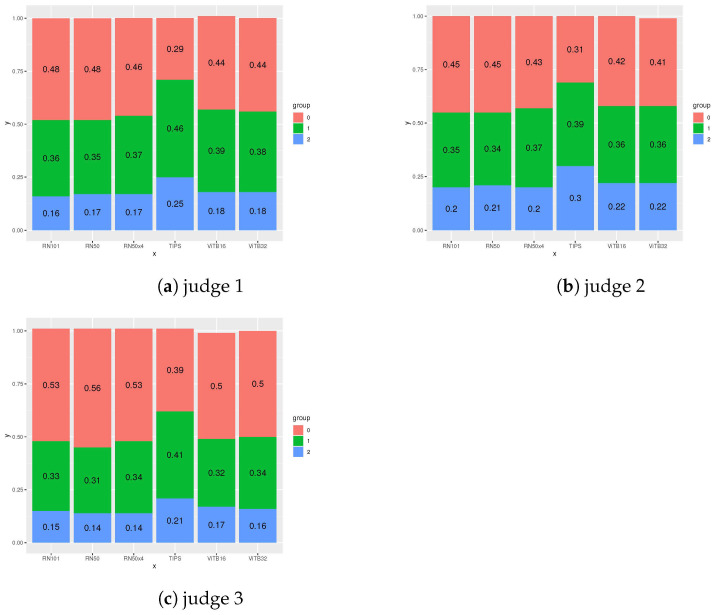
Distribution of JS ratings given by each judge to each descriptive image recommendation algorithm.

For the evaluation based on human judges we used half of all the texts from the Stories426 collection (exactly 1089 one -sentence summaries). We did not conduct the evaluation on the entire dataset because the work of each human judge took several hours and half of the dataset should be sufficient to obtain statistically representative results. In Table 3 and Figure 6, we presented the JS averaged over each illustrative image recommendation algorithm. In Table 4, we also counted the correlation between the JS scores obtained by each recommendation algorithm. In this way, we investigated whether there is a correlation between the scores of each algorithm and the semantic interpretation of their relevance by human judges. To count this correlation matrix, we used the JS scores of all judges simultaneously. In Table 5, we presented the results of the correlation analysis between the JS of the individual judges. We counted this correlation matrix to investigate whether there is a correlation between the scores of individual judges. In Figure 7, we presented the distribution of JS ratings given by each judge to each descriptive image recommendation algorithm.

## 4. Discussion

Unfortunately, we have no way to directly compare the results of the similarity function $sim_{clip}$ computed by each of the algorithms we test, because there are no ground truth values. For this reason, we used the values of $sim_{M_{1,2}}$ and $sim_{M_{1,3}}$ to compare the recommendations of each method.

As can be clearly seen in Equation (11) the TIPS approach with t > 17 has smaller values of $\bar{M_{1,2}}$ and $\bar{M_{1,3}}$ than all other considered algorithms. This fact means that, respectively, the first two or three recommendations given by the TIPS algorithm contain images that are more similar to each other according to the CLIP distance than is the case for algorithms that do not use the voting scheme. According to Figure 4c,d, these values for TIPS decrease exponentially as the parameter t increases. This result should be considered in conjunction with the plot of the values of In(Ξ,t) and Un(Ξ,t) in Figure 4a,b. As can be seen, an increase in the parameter p results in a much slower increase in the common portion of the images recommended by all TIPS component algorithms relative to the sum of the image sets recommended by these component algorithms. This means that each of the component algorithms based on a different image embedding algorithm E that we considered in our study proposed a differentiated set of images $O^{E}{\left(x_{k}\right)}_{t}$. The obtained values of $\bar{M_{1,2}}$, $\bar{M_{1,3}}$, In(Ξ,t), and Un(Ξ,t) indicate that, for the image set we used, the TIPS algorithm allows the integration of the best (highest scored) results of the different recommendation algorithms by diminishing the influence of images that are a disjointed part of the recommendations of the component algorithms. The values of In(Ξ,t) and Un(Ξ,t) increase in a monotonic, near linear fashion.

The shape of the variation of the value of V(Ξ,t) as a function of t is close to logarithmic, which confirms the great diversity when it comes to the recommendations made by the algorithm included in Ξ. We have chosen to highlight a few local maxima that become visible in the spline-smoothed graph and present detailed computations for TIPS at these points in Table 2. Note, however, that the occurrence of these maxima is not universal and may vary depending on the set of illustrative images and text data used. According to the results in Table 2 and Figure 3, the shape of variation of $sim_{min}\left(X\right)$ has a step-function character as the parameter t increases. This means that the recommendation of illustrative images that are among the first t highly scored values returned by the selected component recommendation algorithm might also be recommended by the other component algorithms. This illustrates the situation that, given a certain margin t, the component recommendation algorithms return a similar common portion of the best matching images. The value of $sim_{max}\left(X\right)$ is also spiking, but it reaches its maximum value much faster than $sim_{min}\left(X\right)$. The variability of the coefficients $sim_{med}\left(X\right)$ and $sim_{mean}\left(X\right)$ have an increasing character close to logarithmic and are very similar in shape. They represent some intermediate state between the extreme statistics $sim_{min}\left(X\right)$ and $sim_{max}\left(X\right)$. The entire set of graphs shown in Figure 3 shows that as the parameter p increases, the TIPS algorithm recommends illustrative images with increasing similarity values until it comes to maximising the values of the individual statistics based on $sim_{clip}$. This fact and the shape of plots of $sim_{med}\left(X\right)$ and $sim_{mean}\left(X\right)$ prove that the t parameter is a predictable scaling factor of the confidence range of the recommendation result obtained by TIPS.

Figure 5 visualises examples of the recommendations proposed by the individual component algorithms and the TIPS algorithm for t = 32. Full texts of those stories can be downloaded from https://github.com/JusMia/TIPS/tree/main/stories (accessed on 27 September 2021).

The value t = 32 was chosen because it was the first local maximum V(Ξ,t) for which the values $\bar{M_{1,2}}$ and $\bar{M_{1,3}}$ of TIPS performed better than the individual component algorithms (see Table 1). We can see that each of the illustrative images proposed for texts by the individual component algorithms has some features that make it similar to the given text. An interesting case is sentence (b), in which ViT16 and RN50 seem to be very loosely related to the text and do not capture the semantic meaning of it. It is noteworthy that the TIPS recommendation $O^{Xi}{\left(x_{k}\right)}_{1}$ need not be among the $O^{E}{\left(x_{k}\right)}_{1}$ of the individual component algorithms. The TIPS recommendation t = 32 for sentence (a) is the same as the recommendation for E = ViT16 and E = Vit32 and for sentence (c) for E = RN101. In the case of sentence (b), TIPS t = 32 proposed a different illustrative image, which is semantically consistent with the content of the sentence but not in the set of first recommendations of the individual component algorithms.

The evaluation performed by human judges confirmed that the average JS value is the highest for the TIPS algorithm: see Table 3 and Figure 6. The mean JS for TIPS is 0.96, 0.98 and 0.82 while for a non-voting single-embedding schema (4) with E ViTB32 it was 0.75, 0.81 and 0.66. Also, the results shown in Figure 7 confirm that individual judges are more likely to rate the recommendations made by TIPS as being relevant or highly relevant to the text. Human judges found 25%, 30% and 21% TIPS recommendations highly relevant and 46%, 39% and 41% relevant to text. In the case of non-voting single-embedding schema (4) with E ViTB32 18%, 22% and 16%, recommendations were found highly relevant and 38%, 36% and 34% recommendations were found relevant. According to the results in Table 4, the average value of JS is medium positively correlated between all considered algorithms. Correlation varies from 0.41 to 0.57. This is an important found because it indicates that from the judges perspective all considered algorithms work in similar manner proposing correlated images to subject of the text. As can be seen in Table 5, there is a moderate (0.66 between judge 2 and judge 3) and strong positive correlation (0.76 and 0.77) between judges JS. That means that all three human judges have evaluated algorithms results in a similar manner. The evaluation results showed some limitations of the proposed method due to the fact that it works on a fixed set of image data. In our case the Unsplash Lite dataset nature-themed images might be not suitable for some topics. The judges estimated that between 29% and 39% of the images selected by TIPS were not relevant to the text. However, this is a much better result than when using the non-voting single-embedding schema where the number not relevant to the text was between 41% to even 56%.These results show that using a method based on voting schema allows us to increase the quality of matching evaluated with JS.

## 5. Conclusions

Designing algorithms to suggest illustrative images for text is a very advanced and relatively new research topic. Based on the results presented in the previous section of our paper, we can conclude that our proposed algorithm allows us to make suggestions of illustrative images that are to some extent semantically consistent with the content of the summaries’ sentences of the text to which they relate. The recommendation quality of the TIPS algorithm is a function of the set of illustrative images that have been selected to illustrate the text and the efficiency of sub-recommendation algorithms used by TIPS in the voting schema. An equally important aspect of TIPS is the algorithm that performs text summarization. Since the selection of illustrative images is based on applying a voting schema to the individual sentences included in the summary, this represents a potential weakness in our algorithm if unrepresentative sentences are selected. Regardless, voting schema for individual sentences proved to be a very effective approach. In this paper, we presented a number of coefficients that can be used to test whether a recommendation algorithm $O^{Xi}{\left(x_{k}\right)}_{t}$ meets its expectations. We have done this by presenting some benchmark solutions based on a Stories426 text database and a set of images described in Section 2.2.2.

Based on the evaluation of the results conducted by the three human judges, it can be concluded that the use of the TIPS voting scheme increased the accuracy of matching illustrative images to texts. The judges estimated that the use of TIPS resulted in an increase in matching highly relevant images to text ranging from 5% to 8% and relevant to text ranging from 3% to 7% compared to the approach based on single-embedding schema with E ViTB32, which was the best evaluated algorithm from the single-embedding schema group.

We have shown that the TIPS algorithm allows the integration of the best (highest scored) results of the different recommendation algorithms by diminishing the influence of images that are a disjointed part of the recommendations of the component algorithms. Our computations and experiments can be replicated as we publish the full source codes of both the TIPS algorithm and the entire evaluation process of our approach.

There are many more available image datasets, including common sense ones which could be useful for story understanding in further research including COCO [56] and the Open Images Dataset [57].

An issue that we believe is worthy of future investigation is the feasibility of applying text-based image generation methods at the end of the image processing pipeline. We anticipate that the use of a sufficiently large database of reference images in conjunction with generative adversarial networks (GANs) may allow even better matches of text-illustrating images to be produced [58]. Generating images is an alternative approach to that presented in [59], where a computer method automatically selects already existing images from an album and places them in suitable contexts within a body of text. Using a GAN may result in the ability to generate realistic images instead of picture book-style drawings [60]. It may also be valuable to further validate the resulting illustrative images by re-generating text based on them and comparing it to the original sentences of the summary, for example using the methods described in [61]. Those topics are certainly worthy of further research.

## Figures and Tables

**Table 1:** Coefficient values of $\bar{M_{1,2}}$ and $\bar{M_{1,3}}$ (12) for the recommendation $O^{E}\left(x_{k}\right)$ where embedding E used algorithms ViT16, ViT32 (equivalent to the SBERT method), RN50, RN101, RN50x4, and our proposed TIPS algorithm with *p*-values of 17, 32, 53, and 86.

Method	$\bar{M_{1,2}}$	$\bar{M_{1,3}}$
ViT16	0.017	0.026
ViT32 (SBERT)	0.017	0.026
RN50	0.021	0.032
RN101	0.01	0.015
RN50x4	0.013	0.02
TIPS p=17	0.013	0.03
TIPS p=32	0.008	0.015
TIPS p=53	0.005	0.011
TIPS p=86	0.004	0.008

**Table 2:** The exact values of the coefficients of the TIPS algorithm shown in Figure 3 and Figure 4 for the selected t values.

t	In(Ξ,t)	Un(Ξ,t)	${\mathrm{sim}}_{\min}\left(X\right)$	${\mathrm{sim}}_{\max}\left(X\right)$	${\mathrm{sim}}_{\mathrm{med}}\left(X\right)$	${\mathrm{sim}}_{\mathrm{mean}}\left(X\right)$
1	0.02	4.13	0.40	1.89	0.53	0.65
2	0.05	7.93	0.40	1.91	0.74	0.82
3	0.08	11.69	0.40	1.91	0.86	0.93
17	0.93	60.53	0.55	1.91	1.37	1.35
22	1.33	77.22	0.65	1.91	1.47	1.39
52	4.23	173.77	0.69	1.91	1.53	1.49
86	7.91	279.14	0.79	1.91	1.54	1.52

**Table 3:** The JS averaged over each illustrative image recommendation algorithm.

	Judge 1	Judge 2	Judge 3
TIPS	0.96	0.98	0.82
ViTB32	0.74	0.81	0.66
ViTB16	0.74	0.80	0.67
RN101	0.68	0.75	0.62
RN50x4	0.71	0.77	0.61
RN50	0.69	0.75	0.58

**Table 4:** Correlation between the JS scores obtained by each recommendation algorithm.

	TIPS	ViTB32	ViTB16	RN101	RN50x4	RN50
TIPS	1.00	0.55	0.52	0.55	0.57	0.52
ViTB32	0.55	1.00	0.47	0.46	0.47	0.45
ViTB16	0.52	0.47	1.00	0.44	0.45	0.41
RN101	0.55	0.46	0.44	1.00	0.44	0.44
RN50x4	0.57	0.47	0.45	0.44	1.00	0.48
RN50	0.52	0.45	0.41	0.44	0.48	1.00

**Table 5:** Correlation analysis between the JS of the individual judges.

	Judge 1	Judge 2	Judge 3
judge 1	1.00	0.76	0.77
judge 2	0.76	1.00	0.66
judge 3	0.77	0.66	1.00

## Data Availability

Both source codes and data are available for download from the online repository https://github.com/JusMia/TIPS, accessed on 27 September 2021.
